# Numerical solution of magnetohydrodynamics effects on a generalised power law fluid model of blood flow through a bifurcated artery with an overlapping shaped stenosis

**DOI:** 10.1371/journal.pone.0276576

**Published:** 2023-02-13

**Authors:** Norliza Mohd Zain, Zuhaila Ismail

**Affiliations:** Department of Mathematical Sciences, Faculty of Science, Universiti Teknologi Malaysia (UTM), Johor Bahru, Johor, Malaysia; Tongji University, CHINA

## Abstract

This paper presents a numerical analysis of blood flow in a diseased vessel within the presence of an external magnetic field. The blood flow was considered to be incompressible and fully developed, in that the non-Newtonian nature of the fluid was characterised as a generalised power law model for shear-thinning, Newtonian, and shear-thickening fluids. The impact of a transverse directed external magnetic field on blood flow through a stenosed bifurcated artery was investigated. The arterial geometry was considered as a bifurcated channel with overlapping shaped stenosis. The problem was treated mathematically using the Galerkin Least-Squares (GLS) method. The implementation of this numerical method managed to overcome the numerical instability faced by the classical Galerkin technique when adopted to a highly viscous flow. The benefit of GLS in circumventing the Ladyzhenskaya-Babuška-Brezzi (LBB) condition was utilized by evaluating both the velocity and pressure components at corner nodes of a unstructured triangular element. The non-linearity that emerged from the convective terms was then treated using the Newton-Raphson method, while the numerical integrals were computed using a Gaussian quadrature rule with six quadrature points. The findings obtained from this study were then compared with available results from the literature as well as Comsol multiphysics software to verify the accuracy and validity of the numerical algorithms. It was found that the application of magnetic field was able to overcome flow reversal by 39% for a shear-thinning fluid, 26% for a Newtonian fluid, and 27% for a shear-thickening fluid. The negative pressure and steep wall shear stress which occurs at the extremities of an overlapping stenosis throat were diminished by rise in magnetic intensity. This prevented thrombosis occurrence and produced a uniform calm flow.

## Introduction

Cardiovascular diseases have been reported as one of the primary causes of death among humans worldwide by the World Health Organisation (WHO). Due to this, the genesis, diagnosis, and prognosis of this type of vascular disease has become a favoured subject of scientific research all around the world. Atherosclerotic lesion is an arterial disease that is believed to be responsible for cardiovascular system failure [1]. The plaque formation that accumulates on the inner lining of the artery is made of cholesterol, cellular waste, calcium, fatty substances, and fibrin [2]. The abnormal growth of this plaque formation restricts the normal pattern of blood flow by narrowing the size of the arterial lumen, which then severely reduces the flow of blood to other organs and tissues [3, 4]. Significant blockage of blood circulation could expose an individual to symptoms like pain during exertion (in the chest or legs) caused by atherosclerotic lesions. In most severe cases, the localized plaque may rupture, leading to thrombosis [5]. As the transportation of blood and oxygen through the vessels supplying the heart and brain is gradually obstructed, a heart attack or stroke will occur [6]. Due to the mortality caused by these vascular diseases, researchers are progressively developing mathematical models to simulate and examine the blood flow behaviour caused by stenosis formation. Researchers have also included the influence of various external forces into their models to closer mimic real situations. In term of cost, hemodynamic modelling is a cheap alternative for physicians for predicting the outcome of alternative treatment plans for patients, which can be utilized to predict the risk of disease.

Since blood is a biofluid that is electrical conductive due to the suspension of red blood cells consisting of haemoglobin, which is a form of iron oxide, at a very high concentrations [7], numerous works on Magnetohydrodynamics (MHD) blood flow have been done. Xenos and Tzirtzilakis [39] studied the steady, two-dimensional, incompressible, and laminar flow of blood, which was treated as a Newtonian fluid in a straight artery with a bell shaped stenosis subjected to a uniform magnetic field. This study proved that by reducing the formation of eddies downstream of the stenotic region, the risk of thrombosis was reduced. Sankar et al. [6] conducted research on a cell-free peripheral plasma layer through a particle-fluid suspension core region that flowed through a straight artery with composite shaped stenosis subjected to an external transverse magnetic field, which obtained similar findings to research by Bhatnagar et al. [7], Rabby et al. [8], and Bali and Awasthi [9] for low magnetic field intensity. In contrast, for magnetic field intensity with high or increasing strengths, exposure may lead to untreatable complications such as plaque rupture and vessel wall damage [6]. Hence, the practical application of magnetic field in treating vascular disease works to control blood flow, with careful and thorough steps to prevent irreversible changes to calcium dynamics and skin blood flow. The MHD influence discovered by Misra and Shit [10] was found to be more pronounced on larger diameter arteries compared to smaller diameter arteries. Their findings clarified why magnetic field effects are less effective in a stenotic region in comparison to other regions of a vessel without stenosis. On the other hand, Varshney et al. [11] explored the influence of an externally applied magnetic field with several intensities on laminar, incompressible, and fully developed blood flow, characterized by a generalised power law viscosity model in a straight artery with overlapping shaped stenosis. Their findings showed a decline in the velocity, flow rate, and acceleration of blood flow whilst magnetic field intensity was increased, which is in line with the working principle of MHD.

The MHD principle can be applied to reduce, block, or speed up blood flow in human blood vessels. Thus, it is convenient as a treatment plan for certain cardiovascular diseases, like haemorrhages and hypertension that accelerate blood flow [9]. The production of a Lorentz force through the interaction between magnetic and electric fields to decelerate the movement of fluids is also useful for Magnetic Resonance Imaging (MRI) and blood pumping action [7]. The use of magnets in performing magnetic therapy has also become an alternative medical practice in sports to relieve injuries and pain [12]. Therefore, magnetic field effects on blood flow dynamics has considerable benefits towards biological process, and may provide medical benefits in terms of treatment and magnetic device technology.

The rheological behaviour of blood being considered a non-Newtonian model makes this study more realistic. This is because, within low shear regions, red blood cells are usually found clumped together, forming large particles (rouleaux) [13], causing the viscosity of blood to increase, making the non-Newtonian behaviour of blood more prominent. This phenomenon usually happens in a diseased vessel, downstream of the stenotic region, and in smaller branches and capillaries. Blood behaves differently due to its complex rheological mixture due to its non-Newtonian properties such as shear thinning, viscoelasticity, temperature, as well as its pathological conditions. Since the relation between shear stress and shear rate is non-linear, it is impossible to capture a single constitutive relation that could predict all non-Newtonian behaviours of a fluid flowing in different situations [1, 14, 15]. Extensive studies on different types of non-Newtonian models have been previously discussed with previous authors imitating the complex non-Newtonian characteristics of blood using the generalised power law model [1, 11, 16], Carreau-Yasuda model [17, 18], modified Casson model [19], Cross model [20, 21], and Herschel-Bulkley model [22]. In a study by Ikbal et al. [1], the most general model for non-Newtonian models was found to be the generalised power law model due to its combined properties; being a Newtonian model at low shear rates, a power law model at high shear rates, and a Casson model in special cases. Achab et al. [14] supported Ikbal et al. [1] in the use of the generalised power law model to constitute the non-Newtonian characteristics of blood, since this model includes multiple generalised classical models. Meanwhile, Bakheet et al. [22] opined that in a low shear region, the generalised power-law model can better approximate wall shear stress. By selecting the right non-Newtonian model to predict the wall shear stress values, an accurate prediction of high-risk plaque and blood clots can be achieved. The rheological behaviours of blood flow are described in this paper, based on the study of non-Newtonian fluid models corresponding to bloods shear-thinning and shear-thickening characteristics. A comparison of findings on Newtonian fluid model blood flow characteristics is also reported in this study.

Based on the aforementioned research, there is a quite limited number of the studies on bifurcated arteries despite their physical condition, which consists of junctions, curvatures, and branches that are the most common site for atherosclerotic lesion development. The region along the edge of the daughter artery, as well as downstream of the stenotic region, has a high potential to be distracted by flow disturbances in the form of flow reversal and stagnation [23]. The findings of [23–25] show that the appreciable influence of arterial constriction on blood motion not was limited to flow along the parent artery, but also to flow in the daughter artery. Therefore, arterial geometry should be considered as a bifurcated geometry taken into account in hemodynamics analysis for a thorough understanding of the impacts and risks of atherosclerotic plaque on blood flow along the arterial bifurcation. Because of this, stenosis interpretation should also consider common medical cases. Alsemiry et al. [26] analysed the flow of blood through a straight artery with moderate and severe double stenosis. This is because in real situations, patients are usually diagnosed with multiple stenoses in the same arterial segment. Multiple stenoses are usually form at the femoral and pulmonary arteries [11]. Considering that fact, Varshney et al. [11] and Liu and Liu [27] examined the non-Newtonian flow of blood, using the generalised power law and K-L models, respectively, through a straight artery with overlapping shaped stenosis.

To the best of the author’s knowledge, there has been no study on the MHD flow of non-Newtonian blood generalised through the power law model in a bifurcated artery with an arterial constriction at the mother artery in the shape of overlapping in the literature. Hence, in this study, we are interested in the mathematical modelling of the generalised power law fluid model for blood flow through a stenosed bifurcated artery under the action of an externally applied uniform magnetic field. In our previous work [28], MHD effect was examined using a Newtonian model that possessed a constant viscosity. To utilise the benefit of the Galerkin least-squares method in overcoming the inherent instability arising from the approximation of highly advective dominated flows [29], we decided to implement this method to solve the shear dominated flow of blood subjected to the MHD effect. As reported in [29–34], the GLS method had successfully generated stable and converged approximations for original Galerkin finite element formulations through stability parameters made from residual-based terms. Hence, through GLS implementation, all undesirable pathologies experienced in an original Galerkin formulation could be overcome, including the requirement to satisfy the LBB condition in achieving a compatible combination of velocity and pressure subspaces [31–33]. The GLS formulation was applied to various kinds of non-Newtonian fluids in [29–34] but was limited to flow over a lid-driven cavity, 4:1 sudden expansion, and 4:1 sudden contraction. Hence, we believe there is a huge potential for extending the cited references on the hemodynamic behaviours of blood through bifurcated channels. The problem investigated here involves the shear-thinning, Newtonian, and shear-thickening natures of blood flow, which are classified according to different generalised power law indexes. In this study, the computational domain of a bifurcated artery that possessed overlapping shaped stenosis at a mother artery was discretized using an unstructured linear triangular element with equal order of interpolation functions for both velocity and pressure.

## Mathematical formulation

### Governing equations and boundary conditions

In this study, blood was modelled as an incompressible fluid that exhibited non-Newtonian properties flowing through a bifurcated artery with an overlapping shaped stenosis located in the mother artery. The flow of blood in the arterial bifurcation was considered laminar and fully developed. The flow of blood was subjected to a uniform magnetic field, *B*_0_ that was applied in the axial direction. Hence, the governing equations for such flow conserved mass and momentum as follows:

$$\begin{array}{c} \begin{array}{c} \nabla \cdot \bar{u}=0\;\text{in}\;\bar{\Omega },\\ \rho \bar{u}\cdot \nabla \bar{u}-\nabla \cdot \bar{\tau }+\nabla \bar{p}=\rho \bar{f}\;\text{in}\;\bar{\Omega }, \end{array} \end{array}$$ (1)

where $\bar{u}$ is the velocity component, *ρ* is the blood density, $\bar{\tau }$ is the stress tensor, $\bar{p}$ is the pressure or volumetric stress, $\bar{f}$ is the dimensional body force vector, and $\bar{\Omega }$ represents the domain in dimensional form. Due to the assumption of unidirectional flow velocity in in an axial direction, the body force vector $\bar{f}$ which is comprised of the term $-\sigma B_{0}^{2}\bar{u}$, appears on the left hand side of the *x*-momentum equation. This term represents the Lorentz force per unit volume, which arises due to the fluid’s electrical conductivity, *σ*. The parameter *B*_0_ indicates magnetic flux intensity, which is applied perpendicularly to the direction of fluid flow. As mentioned before, in this study, we considered the use of the generalised power law model, which is a form of Generalized Newtonian Liquid (GNL) that allows viscosity to vary with shear rate to represent the rheological behaviour of blood flow. According to [29, 35], by definition in the generalized Newtonian liquid model, the stress tensor is given as a constitutive equation which comprises the sum of volumetric stress and viscous stress components that takes the form:

$$\begin{array}{c} \bar{\tau }=-\bar{p}I+2\bar{\eta }\left(\dot{\gamma }\right)D, \end{array}$$ (2)

where **I** is the unit tensor, $\bar{\eta }\left(\dot{\gamma }\right)$ is the viscosity function, and **D** is the strain rate tensor which may be defined as:

$$\begin{array}{c} D=\frac{1}{2}(\nabla \bar{u}+{\left(\nabla \bar{u}\right)}^{T}). \end{array}$$ (3)

The non-Newtonian viscosity function, $\bar{\eta }\left(\dot{\gamma }\right)$ depends on the scalar $\dot{\gamma }$, which acts as the magnitude of the strain rate tensor given by the expression:

$$\begin{array}{c} \dot{\gamma }=\sqrt{2D\left(\bar{u}\right):D\left(\bar{u}\right)}. \end{array}$$ (4)

GNL constitutive equation allows fluid viscosity to vary with shear rate. In this model, non-Newtonian fluid behaviour can be interpreted based on the stress-strain rate relationship in terms of the shear-thinning and shear-thickening natures of the fluid. Hence, for the generalised power law model, the viscosity function, $\bar{\eta }\left(\dot{\gamma }\right)$ that is employed in Eq 2 may be defined as:

$$\begin{array}{c} \bar{\eta }\left(\dot{\gamma }\right)=-m{\left|\left(I_{2}\right)\right|}^{\frac{\left(n-1\right)}{2}}, \end{array}$$ (5)

where *m* represents the fluid consistency parameter, *I*_2_ is the second invariant of the strain rate tensor, and *n* is the power law index. When the viscosity function $\bar{\eta }\left(\dot{\gamma }\right)$ in Eq 5 is represented by a constant value of *μ*, Eq 2 reduces to the classical Newtonian viscosity model. In this study, three cases of fluid viscosities was considered, which were pseudoplastic, Newtonian, and dilatant fluids. Pseudoplastic fluid, better known as shear-thinning fluid, is a fluid whose viscosity decreases as the shear rate is increased through the parameter *n* < 1. Then the Newtonian fluid with constant viscosity, *μ* is obtained at *n* = 1. Meanwhile, dilatant fluid behaves as a shear thickening fluid in that *n* > 1 represents increased fluid viscosity as shear rate increases. For two dimensional flow, the explicit form of *I*_2_ is given as:

$$\begin{array}{c} I_{2}=2{\left(\frac{\partial \bar{u}}{\partial \bar{x}}\right)}^{2}+2{\left(\frac{\partial \bar{v}}{\partial \bar{y}}\right)}^{2}+{\left(\frac{\partial \bar{u}}{\partial \bar{y}}+\frac{\partial \bar{v}}{\partial \bar{x}}\right)}^{2}. \end{array}$$ (6)

Hence, by using the vector notation, the governing equations for the generalized Newtonian liquid in a domain $\bar{\Omega }\subset R^{2}$ are given as [29, 33]:

$$\begin{array}{c} \begin{array}{c} \nabla \cdot \bar{u}=0\;\text{in}\;\bar{\Omega },\\ \rho \bar{u}\cdot \nabla \bar{u}-2\bar{\eta }\left(\dot{\gamma }\right)\nabla \cdot D+\nabla \bar{p}=\rho \bar{f}\;\text{in}\;\bar{\Omega }. \end{array} \end{array}$$ (7)

The boundary conditions prescribed along the computational domain are specified as [36]:

$$\begin{array}{c} \begin{array}{cc} & \text{Inflow:}\;\bar{u}\left(0,\bar{y}\right)=\frac{3}{2}{\bar{u}}_{r}(1-{\left(\frac{\bar{y}}{a}\right)}^{\frac{n+1}{n}}),\;\bar{v}\left(0,\bar{y}\right)=0,\\ & \text{Outflow:}\;\left(-\bar{p}I+2\bar{\eta }\left(\dot{\gamma }\right)D\right)n={\bar{t}}_{h},\;\text{where}\;{\bar{t}}_{h}=0,\;\text{on}\;{\bar{\Gamma }}_{h},\\ & \text{Outer}\;\text{and}\;\text{inner}\;\text{wall:}\;\bar{u}=0,\;\text{on}\;{\bar{\Gamma }}_{g},\\ & \text{Pressure:}\;\bar{p}\left(\bar{x},\bar{y}\right)=0,\;\text{at}\;\left(0.06\text{m},0.017349\text{m}\right)\;\text{and}\;\left(0.056175\text{m},-0.0239595\text{m}\right). \end{array} \end{array}$$ (8)

In Eq 8, the parameters ${\bar{\Gamma }}_{g}$ and ${\bar{\Gamma }}_{h}$ denote part of the boundary $\bar{\Gamma }$ on the domain $\bar{\Omega }$ where Dirichlet and Neumann conditions are, respectively, imposed. Other than that, the reference velocity, ${\bar{u}}_{r}$ represents the average mean inflow velocity, **n** is the unit outward normal vector, and ${\bar{t}}_{h}$ is the vector of the prescribed boundary tractions. Note that the bar symbol on top of each variable demonstrates the dimensional form of these quantities.

### Computational domain

To perform mathematical analysis for the problem under consideration, the following assumptions was inflicted onto the artery geometry:

1. The artery forming bifurcation is of finite length.
2. The parent aorta possesses a single overlapping shaped stenosis in its lumen.
3. Curvatures are introduced at the lateral junctions and the flow divider of the arterial bifurcation to ensure that one can rule out the presence of any discontinuity causing non-existent separation zones.

The geometry of the stenosed bifurcated artery considered in this study was modelled as a bifurcated channel as proposed by Chakravarty and Mandal [37]. Instead of applying a mild shaped stenosis, overlapping shaped stenosis was applied, which was constructed mathematically by Chakravarty and Mandal [38].

The computational domain considered herein was constructed using the two-dimensional Cartesian coordinate system. Due to that, the dimensional coordinates of a material point $\left(\bar{x},\bar{y}\right)$ denote the horizontal axis of the trunk and radial direction, respectively. Meanwhile, the origin of the axis is taken as a point which falls asymmetrically on the inlet of the arterial. The geometry of the bifurcated artery with overlapping shaped stenosis may be set up mathematically when the outer wall geometry is described by:

$$\begin{array}{c} \bar{R_{1}}\left(\bar{X}\right)=\{\begin{array}{cc} & a,\;0\leq \bar{x}\leq d\;\text{and}\;l_{0}\leq \bar{x}\leq x_{1},\\ & a-\frac{3\tau_{m}}{2l_{0}^{4}}\{\begin{array}{c} 11\left(x-d\right)l_{0}^{3}-47{\left(x-d\right)}^{2}l_{0}^{2}\\ +72{\left(x-d\right)}^{3}l_{0}-36{\left(x-d\right)}^{4} \end{array}\},\;d\leq \bar{x}\leq d+l_{0},\\ & a+r_{0}-\sqrt{r_{0}^{2}-{\left(x-x_{1}\right)}^{2}},\;x_{1}\leq \bar{x}\leq x_{2},\\ & 2r_{1}\;\text{sec}\;\beta +\left(x-x_{2}\right)\;\text{tan}\;\beta ,\;x_{2}\leq \bar{x}\leq x_{max}-s. \end{array} \end{array}$$ (9)

In addition, the inner wall geometry is constructed as:

$$\begin{array}{c} \bar{R_{2}}\left(\bar{X}\right)=\{\begin{array}{cc} & 0,\;0\leq x\leq d\;\text{and}\;0\leq \bar{x}\leq x_{3},\\ & \sqrt{r_{0}^{\prime }-{\left(x-\left(x_{3}+r_{0}^{\prime }\right)\right)}^{2}},\;x_{3}\leq \bar{x}\leq x_{4},\\ & r_{0}^{\prime }\;\text{cos}\;\beta +\left(x-x_{4}\right)\;\text{tan}\;\beta ,\;x_{4}\leq \bar{x}\leq x_{max}, \end{array} \end{array}$$ (10)

where $\bar{R_{1}}\left(\bar{X}\right)$ and $\bar{R_{2}}\left(\bar{X}\right)$ represent the respective radii of the outer and inner wall of the bifurcated artery in the dimensional form, respectively. Meanwhile, the respective radii of the mother and daughter artery are represented by *a* and *r*_1_, respectively. The curvature radii for the lateral junction and the flow divider are indicated by parameters *r*_0_ and $r_{0}^{\prime }$, respectively. Whereas the length of the stenosis is characterised as *l*_0_, which is measured at a distance *d* from the origin. The location for the onset and offset of the lateral junction are denoted, respectively, by *x*_1_ and *x*_2_. The apex of the branch artery is indicated by *x*_3_. Meanwhile, *τ*_*m*_ acts as the maximum height of the overlapping stenosis that occurs at $d+\frac{2l_{0}}{6}$ and $d+\frac{4l_{0}}{6}$. The half-angle of the branch artery is represented as *β*. Parameters involved in the above expressions 9 and 10 can be specified as:

$$\begin{array}{c} \begin{array}{cc} & x_{2}=x_{1}+r_{0}\;\text{sin}\;\beta ,\\ & r_{0}=\frac{a-2r_{1}\;\text{sec}\;\beta }{\;\text{cos}\;\beta -1},\\ & r_{0}^{\prime }=\frac{(x_{3}-x_{2})\;\text{sin}\;\beta }{1-\;\text{sin}\;\beta },\\ & x_{3}=x_{2}+q,\\ & s=2r_{1}\;\text{sin}\;\beta ,\\ & x_{4}=x_{3}+r_{0}^{\prime }\left(1-\;\text{sin}\;\beta \right). \end{array} \end{array}$$

Parameter *q* is a small number that is chosen for geometry compatibility, which lies in the range of 0.0001 ≤ *q* ≤ 0.0005. The schematic diagram for the geometry of the bifurcated channel in the presence of overlapping shaped stenosis constructed mathematically using Eqs 9 and 10 are presented in the next section in non-dimensional form.

## Numerical methodology

### Non-dimensionalisation procedure

**Fig 1:**
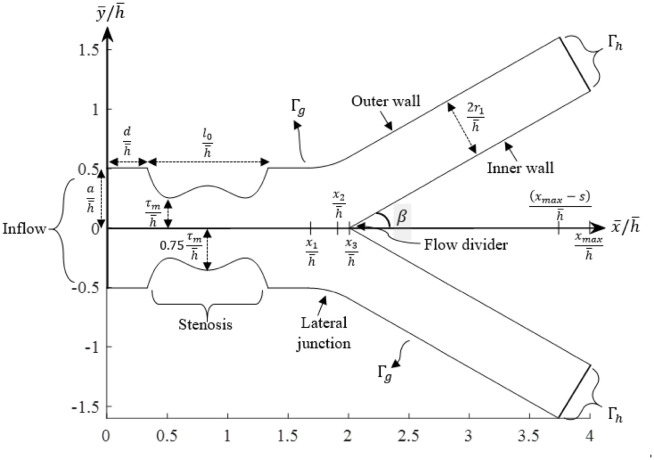
Geometry of the stenosed arterial bifurcation model.

To simplify the physical dimensions from the dimensional computational domain constructed using Eqs 9 and 10, the governing equations defined in Eq 7 were subjected to appropriate boundary conditions as specified in Eq 8. The following non-dimensional variables were later introduced for parameter rescaling as follows:

$$\begin{array}{c} \begin{array}{cc} & x=\frac{\bar{x}}{\bar{h}},\;y=\frac{\bar{y}}{\bar{h}},\;u=\frac{\bar{u}}{{\bar{u}}_{r}},\;v=\frac{\bar{v}}{{\bar{u}}_{r}},\;p=\frac{\bar{p}}{\rho {\bar{u}}_{r}^{2}},\;\eta \left(\dot{\gamma }\right)=\frac{\bar{\eta }\left(\dot{\gamma }\right){\bar{h}}^{n-1}}{m{\bar{u}}_{r}^{n-1}}, \end{array} \end{array}$$ (11)

where $\bar{x}$ and $\bar{y}$ denote the axis of a point that is taken along the axis of the horizontal and radial direction, respectively. The characteristic length, $\bar{h}$ refers to the length of the inlet, which is equal to 2*a*. Byy using the transformation variables introduced in Eq 11, the boundary configuration of the computational domain, Ω was non-dimensionalised to a horizontal length of 4 with a vertical inlet equal to 1 as shown in Fig 1:

Since this study concerns the MHD effect of the generalised power law model of blood flow, by the non-dimensionalisation procedure, two dimensionless parameters of Reynolds and Hartmann numbers was respectively attained as:

$$\begin{array}{c} \text{Re}=\frac{\rho {\bar{h}}^{n}}{m{\bar{u}}_{r}^{n-2}},\;\text{and}\;M=B_{0}{\left(\frac{\sigma {\bar{h}}^{n+1}}{m{\bar{u}}_{r}^{n-1}}\right)}^{\frac{1}{2}}. \end{array}$$ (12)

From this transformation, the effective viscosity from Eq 5 was non-dimensionalised to:

$$\begin{array}{c} \eta \left(\dot{\gamma }\right)=|2{\left(\frac{\partial u}{\partial x}\right)}^{2}+2{\left(\frac{\partial v}{\partial y}\right)}^{2}+{\left(\frac{\partial u}{\partial y}+\frac{\partial v}{\partial x}\right)}^{2}{|}^{\frac{n-1}{2}}. \end{array}$$ (13)

By the substitution of all the non-dimensional parameters introduced in Eq 11 to the governing equations stated in Eq 7, the following governing equations were obtained in a non-dimensional form as follows:

$$\begin{array}{c} \begin{array}{c} \nabla \cdot u=0\;\text{in}\;\Omega ,\\ u\cdot \nabla u-\frac{1}{\text{Re}}2\eta \left(\dot{\gamma }\right)\nabla \cdot D+\nabla p=f\;\text{in}\;\Omega . \end{array} \end{array}$$ (14)

Furthermore, the boundary conditions in Eq 8 was simplified to:

$$\begin{array}{c} \begin{array}{cc} & \text{Inflow:}\;u\left(x,y\right)=\frac{3}{2}(1-{\left(\frac{y}{0.5}\right)}^{\frac{n+1}{n}}),\;v\left(x,y\right)=0,\;\text{at}\;(x=0,-0.5\leq y\leq 0.5),\;\\ & \text{Outflow:}\;\left(-pI+\frac{1}{\text{Re}}2\eta \left(\dot{\gamma }\right)D\right)n=t_{h},\;\text{where}\;t_{h}=0,\;\text{on}\;\Gamma_{h},\\ & \text{Outer}\;\text{and}\;\text{inner}\;\text{wall:}\;u=0,\;\text{on}\;\Gamma_{g},\\ & \text{Pressure:}\;p(x,y)=0,\;\text{at}\;(4,1.1556)\;\text{and}\;(3.745,-1.5973). \end{array} \end{array}$$ (15)

From the similarity transformation, the dimensional force vector $\bar{f}$ in Eq 1 was simplified to the dimensionless force vector **f**, which was obtained as $-\frac{M^{2}}{\text{Re}}u$, where *M* indicates the magnetic field intensity applied in a transverse direction. The above governing equations were solved in two domains. The first domain was taken from the study conducted by Xenos and Tzirtzilakis [39] which is a straight channel with bell shaped stenosis, which served as a test domain to verify that the source code developed in Matlab would work according to the GLS algorithms. Then, as the validity of the developed algorithms was obtained, similar algorithms were implemented in the second domain of interest, which is a bifurcated channel with constriction portrayed as an overlapping shape.

### Galerkin least-square formulation

The Galerkin least-squares method is known as a stabilized form of the finite element method, and was developed to enhance the stability of mixed or classical Galerkin FEM in solving fluid flow problems involving an incompressible and highly viscous flow [40]. The GLS method was developed by adding perturbation terms originating from the least-squares of the momentum residuals to the classical Galerkin FEM formulation [30, 40]. To preserve the consistency of the method at an optimal convergence, the added terms must be mesh-dependent. To approximate the velocity and pressure components in Eq 14 a Galerkin Least-Squares (GLS) method based on the usual fluid dynamics concept was used with finite element subspaces for velocity and pressure fields over a finite element partition *C*_*h*_ of the closed domain Ω following [31]:

$$\begin{array}{c} \begin{array}{c} V_{h}=\{N\in {\left[H_{0}^{1}\left(\Omega \right)\right]}^{2}\;|\;N_{|K}\in R_{1}{\left(K\right)}^{2},\;K\in C_{h}\},\\ V_{h}^{g}=\{N\in {\left[H^{1}\left(\Omega \right)\right]}^{2}\;|\;N_{|K}\in R_{1}{\left(K\right)}^{2},\;K\in C_{h},\;N=u_{g}\;\text{on}\;\Gamma_{g}\},\\ P_{h}=\{p\in C^{0}\left(\Omega \right)\cap L_{0}^{2}\left(\Omega \right)\;|\;Q_{|K}\in R_{1}{\left(K\right)}^{2},\;K\in C_{h}\}. \end{array} \end{array}$$ (16)

Based on the approximation functions defined in Eq 16, the Galerkin least-squares formulation for boundary value problem introduced in Eq 14 were subjected to the boundary conditions specified in Eq 15 as:

To find the pair of (**u**_*h*_, *p*_*h*_) in the same vector space of (**V**_*h*_ × *P*_*h*_) for all (**N**, *q*) also in the same vector space, such that:

$$\begin{array}{c} B\left(u_{h},p_{h};N,q\right)=F\left(N,q\right),\;\forall \left(N,q\right)\in \left(V_{h}\times P_{h}\right), \end{array}$$ (17)

where

$$\begin{array}{c} \begin{array}{cc} B(u,p;N,q) & =\int_{\Omega }\left[\text{grad}u\right]u\cdot Nd\Omega +\int_{\Omega }\frac{1}{\text{Re}}2\eta \left(\dot{\gamma }\right)D\left(u\right)\cdot D\left(N\right)d\Omega \\ & -\int_{\Omega }p\text{div}Nd\Omega -\int_{\Omega }q\text{div}ud\Omega +\\ & +\sum_{\Omega_{K\in C_{h}}}\int_{\Omega_{K}}[\begin{array}{c} ([\text{grad}u]u+\text{grad}p\\ -\text{div}(\frac{1}{\text{Re}}2\eta \left(\dot{\gamma }\right)D\left(u\right)))\\ \cdot \tau \left({\text{Re}}_{K}\right)(\left[\text{grad}N\right]u-\text{grad}q\\ -\text{div}(\frac{1}{\text{Re}}2\eta \left(\dot{\gamma }\right)D\left(N\right))) \end{array}]d\Omega_{K}, \end{array} \end{array}$$ (18)

$$\begin{array}{c} \begin{array}{cc} F(N,q) & =\int_{\Omega }f\cdot Nd\Omega +\int_{\Gamma }t\cdot Nd\Gamma +\\ & +\sum_{\Omega_{K\in C_{h}}}\int_{\Omega_{K}}f\cdot \tau \left({\text{Re}}_{K}\right)[\begin{array}{c} ([\text{grad}N]u-\text{grad}q\\ -\text{div}(\frac{1}{\text{Re}}2\eta \left(\dot{\gamma }\right)D\left(N\right))) \end{array}]d\Omega_{K}. \end{array} \end{array}$$ (19)

The terms involved in Eqs 18 and 19 were counted elementwise, with positive parameter *τ*(Re_*K*_) acting as a stabilization parameter throughout this study. The process of constructing *τ*(Re_*K*_) parameters is not unique [40]. These parameters are positive coefficients developed through either estimation of error, dimensional analysis, or convergence proof [40]. The stability parameters were designed in this study as a function of the element Reynolds number with the ratio of element size to velocity scale defined according to [29, 32] as follows:

$$\begin{array}{c} \begin{array}{cc} \tau ({\text{Re}}_{K}) & =\frac{h_{K}}{{2|u|}_{p}}\xi \left(Re_{K}\right),\\ {\text{Re}}_{K} & =\frac{m_{K}\rho {\left|u\right|}_{p}h_{K}}{4\eta (\dot{\gamma })},\\ \xi (Re_{K}) & =\{\begin{array}{cc} & Re_{K},\;0\leq {\text{Re}}_{K}<1,\\ & 1,\;Re_{K}\geq 1, \end{array}\\ m_{K} & =\text{min}\{\frac{1}{3},2C_{K}\},\\ \;h_{K}{\left\|\Delta N\right\|}_{0,K}^{2} & \leq C_{K}{\left\|\nabla N\right\|}_{0}^{2},\;\forall N\in V_{h}. \end{array} \end{array}$$ (20)

From Eqs 18 and 19, it is clear that the terms before summation originate from the classical Galerkin formulation. Meanwhile, the terms within the summation sign are the stabilized terms added to the classical Galerkin formulation to generate a stable approximation, thus Eqs 18 and 19 resulted in an GLS formulation as a whole. The terms *h*_*K*_ acted as the element size, while the *p* norm expression ${\left|u\right|}_{p}=(\sum_{i=1}^{2}|u_{i}{{|}^{2})}^{\frac{1}{2}}$ was defined according to [30, 41]. As for the constants *C*_*K*_, they had been derived previously in [42] through inverse estimation of an advection-diffusion equation, which indicated *C*_*K*_ = ∞ for linear element *P*_1_/*P*_1_ and *C*_*K*_ = 1/48 for quadratic element *P*_2_/*P*_2_. The discretization of Eqs 17–20 was performed by substituting the trial and weighting functions **u**, *p* and **N**, *q* in terms of their finite element expansions for piecewise linear triangular elements *P*_1_/*P*_1_. Due to this, the spatial variations for the velocity and pressure within an element *e*_*i*_, *i* = 1, 2, 3, …, *N*_*e*_ were approximated as:

$$\begin{array}{c} \begin{array}{cc} & u=u^{e_{i}}=\sum_{j=1}^{3}N_{j}^{e_{i}}u_{j}^{e_{i}}=Nu,\\ & p=p^{e_{i}}=\sum_{j=1}^{3}q_{j}^{e_{i}}p_{j}^{e_{i}}=qp, \end{array} \end{array}$$ (21)

where *j* = 1, 2, 3 corresponds to three corner nodes per element *e*_*i*_. The use of an iterative technique called the Newton-Raphson method was required to linearize the non-linear term in the dependent variables presented in the non-linear system of Eqs 18 and 19. This non-linear term was derived from the momentum equations as advection terms. To implement this iterative technique, the variational problem in Eqs 18 and 19 needed to be written in terms of their residual functional form, **R**(**U**), which is given as:

$$\begin{array}{c} R(U)=[K(U)]U-\{F\}=0, \end{array}$$ (22)

where **U** represents the vector for the degrees of freedom of **u** and *p*, [**K**(**U**)] stands for the stiffness matrices comprised of the advective and diffusive matrices as well as the incompressibility constraints, and {**F**} denotes the force vector. To provide clarity the Newton-Raphson method execution was used to solve the two-dimensional, incompressible, steady, and laminar flow of blood subjected to the magnetohydrodynamic effects in the form of **U** = [**u**_**1**_, **u**_**2**_, *and* **p**], respectively. The components for the vector **R**(**U**) were later detailed as follows:

$$\begin{array}{c} \begin{array}{cc} R_{1} & =C_{1}\left(u_{1}\right)u_{1}+C_{2}\left(u_{2}\right)u_{1}-Q_{1}p+\frac{1}{\text{Re}}[K_{11}+K_{22}]u_{1}\\ & +(G_{1}^{\tau }\left(u_{2}\right)+G_{2}^{\tau }\left(u_{1}\right))u_{1}+M_{11}^{\tau }\left(p\right)u_{1}+M_{12}^{\tau }\left(p\right)u_{2}-F_{12},\\ R_{2} & =C_{1}\left(u_{1}\right)u_{2}+C_{2}\left(u_{2}\right)u_{2}-Q_{2}p+\frac{1}{\text{Re}}[K_{11}+K_{22}]u_{2}\\ & +(G_{1}^{\tau }\left(u_{2}\right)+G_{2}^{\tau }\left(u_{1}\right))u_{2}+M_{21}^{\tau }\left(p\right)u_{1}+M_{22}^{\tau }\left(p\right)u_{2}-F_{21},\\ R_{3} & =-T_{1}u_{1}-T_{2}u_{2}-T_{1}^{\tau }\left(u_{1}\right)u_{1}-T_{1}^{\tau }\left(u_{2}\right)u_{1}-T_{2}^{\tau }\left(u_{1}\right)u_{2}\\ & -T_{2}^{\tau }\left(u_{2}\right)u_{2}-T_{11}^{\tau }p-T_{22}^{\tau }p, \end{array} \end{array}$$ (23)

where,

$$\begin{array}{c} \begin{array}{cc} C_{w}\left(v_{w}\right) & =\int_{\Omega_{e}}N^{T}\left(Nu_{w}\right)\frac{\partial N}{\partial x_{w}}d\Omega_{e},\;Q_{r}=\int_{\Omega_{e}}\frac{\partial N^{T}}{\partial x_{r}}qd\Omega_{e},\\ K_{rw} & =\eta \left(\dot{\gamma }\right)\int_{\Omega_{e}}\frac{\partial N^{T}}{\partial x_{r}}\frac{\partial N}{\partial x_{w}}d\Omega_{e},\;T_{r}=\int_{\Omega_{e}}q^{T}\frac{\partial N}{\partial x_{r}}d\Omega_{e},\\ G_{r}^{\tau }\left(u_{w}\right) & =\sum_{\Omega_{K_{e}\in C_{h}}}\int_{\Omega_{K_{e}}}\tau \left(Re_{K}\right)Nu_{w}\frac{\partial N^{T}}{\partial x_{w}}(Nu_{r}\frac{\partial N}{\partial x_{r}}+Nu_{w}\frac{\partial N}{\partial x_{w}})d\Omega_{K_{e}},\\ M_{rw}^{\tau }\left(p\right) & =\sum_{\Omega_{K_{e}\in C_{h}}}\int_{\Omega_{K_{e}}}\tau \left(Re_{K}\right)\frac{\partial q}{\partial x_{r}}p(\frac{\partial N^{T}}{\partial x_{w}}N)d\Omega_{K_{e}},\\ T_{r}^{\tau }\left(u_{w}\right) & =\sum_{\Omega_{K_{e}\in C_{h}}}\int_{\Omega_{K_{e}}}\tau \left(Re_{K}\right)\frac{\partial q^{T}}{\partial x_{r}}(Nu_{w}\frac{\partial N}{\partial x_{w}})d\Omega_{K_{e}},\\ T_{\text{rw}}^{\tau } & =\sum_{\Omega_{K_{e}\in C_{h}}}\int_{\Omega_{K_{e}}}\tau \left(Re_{K}\right)\frac{\partial q^{T}}{\partial x_{r}}\frac{\partial q}{\partial x_{w}}d\Omega_{K_{e}},\\ F_{\text{rw}} & =\oint_{\Gamma_{e}}t_{r}\cdot N^{T}d\Gamma_{e}+\int_{\Omega_{e}}f_{r}\cdot N^{T}d\Omega_{e}+\\ & +\sum_{\Omega_{K_{e}\in C_{h}}}\int_{\Omega_{K_{e}}}f_{r}\cdot \tau \left({\text{Re}}_{K}\right)[\begin{array}{c} u_{r}\frac{\partial N^{T}}{\partial x_{r}}+u_{w}\frac{\partial N^{T}}{\partial x_{w}}-\frac{\partial q^{T}}{\partial x_{r}} \end{array}]d\Omega_{K_{e}}. \end{array} \end{array}$$ (24)

The traction boundary conditions, written in Eq 23 are the first terms of parameters **F**_**12**_ and **F**_**21**_, which represent the surface integrals of the force vector that is induced naturally from Green’s theorem. These expressions are made of combinations of hydrostatic boundary stress and the total boundary stress [40]. Besides, the parameters **F**_**12**_ and **F**_**21**_ also comprise the body force vector, **f**_**r**_ which is equal to $\left(-\frac{M^{2}}{\text{Re}}Nu_{r},0\right)$, respectively, for (**f**_**1**_, **f**_**2**_). It is important to note that the stabilization terms presented in Eqs 18 and 19 involving viscous diffusion terms reduces to zero due to a low order element. Furthermore, to complete the linearization procedure in this study, a Jacobian matrix **J**(**U**) was also derived by differentiating terms from Eq 23 that were non-linear dependent variables as defined by:

$$\begin{array}{c} J\left(U\right)=\frac{\partial R(U)}{\partial U}=[\begin{array}{ccc} \frac{\partial R_{1}}{\partial u_{1}} & \frac{\partial R_{1}}{\partial u_{2}} & \frac{\partial R_{1}}{\partial p}\\ \frac{\partial R_{2}}{\partial u_{1}} & \frac{\partial R_{2}}{\partial u_{2}} & \frac{\partial R_{2}}{\partial p}\\ \frac{\partial R_{3}}{\partial u_{1}} & \frac{\partial R_{3}}{\partial u_{2}} & \frac{\partial R_{3}}{\partial p} \end{array}]. \end{array}$$ (25)

Due to this, the Jacobian entries in Eq 25 were derived using combinations of the original entries from Eq 23, plus a few new terms that emerged from the differentiation of the residual function, $\frac{\partial R(U)}{\partial U}$. Hence, according to the Newton-Raphson method, the solutions for degrees of freedom were obtained by solving:

$$\begin{array}{c} U^{b+1}=U^{b}-J^{-1}\left(U^{b}\right)R\left(U^{b}\right), \end{array}$$ (26)

where the superscript *b* represents the iteration. For this study, the convergence criteria *τ* of a maximum residual was employed at 10^−4^, which was computed as:

$$\begin{array}{c} \tau \leq \sqrt{\sum_{I=1}^{N}{\left(R_{I}\left(U^{b}\right)\right)}^{2}}. \end{array}$$ (27)

Meanwhile, the numerical integrals were approximated using the Gaussian quadrature techniques of six quadrature points. Based on the GLS formulations explained before, the following algorithms were implemented in the developed source code by using the Matlab programming software:

(i) The domain geometry is constructed as shown in Fig 1. Finite element meshes are then generated from a “mesh2d” function developed by Darren Engwirda, which is shared online and can be downloaded from the MathWorks webpage (www.mathworks.com).
(ii) The boundary conditions, convergence tolerance, *τ* and all related parameters values are consequently prescribed. The initial guess for **U**^**0**^ is generated from the Stokes solution, where *n* is made equal to 1 (Newtonian fluid).
(iii) Element matrix is evaluated for each finite element, where parameter *n* is defined according to the rheological behaviour of fluid it is supposed to represent with the integrals computed using a Gaussian quadrature rule, with the stabilization GLS terms calculated from Eq 20.
(iv) Element matrices are then being assembled to form a global matrix, and later solved, subjected to the boundary conditions as stated in Eq 15.
(v) The residual **R(U)** components in Eq 23 is later approximated.
(vi) Convergence tolerance, *τ* as defined in Eq 27 is evaluated to check for the convergence of the solution. When this condition is fulfilled, the iteration will stop, and the solutions should be obtained in the previous step, *b*.
(vii) If the previous condition (vi) has not been satisfied, the Jacobian matrix **J(U)** should be evaluated as specified in Eq 25.
(viii) The solutions should be corrected as **U**^*b*+1^ = **U**^*b*^ + *δ* **U**^*b*^ with *δ* **U**^*b*^ appearing to be *δ* **U**^*b*^ = −**J**^−1^(**U**^*b*^)**R**(**U**^*b*^), which is the second term on the right hand side of Eq 26.

As the solution of **U** = [**u**_**1**_, **u**_**2**_, **p**] is acquired in the last iteration where the limit as stated in Step (vi) is fulfilled, the iteration will stop. The solution obtained for several important variables such as the wall shear stress, wall pressure, and velocity are visualized graphically. Wall shear stress is the friction that produces a tangential force exerted by the blood flowing across the vessel wall, which is calculated as:

$$\begin{array}{c} \tau_{w}=m\eta \left(\dot{\gamma }\right)(\frac{\partial u}{\partial y}+\frac{\partial v}{\partial x}){|}_{y=R_{1}\left(x\right)}. \end{array}$$ (28)

## Numerical validations

Numerical validation was performed to validate that the source code developed using Matlab software was properly programmed according to the GLS algorithm. The developed source code was verified through a result comparison of Xenos and Tzirtzilakis [39] who treated the flow of blood as a Newtonian fluid model flowing through a straight channel in the presence of bell-shaped stenosis. Apart from that, a comparison of results was also made with the results computed using Comsol Multiphysics 5.2 software. Problems considered by Xenos and Tzirtzilakis were solved numerically using the Semi-Implicit Method For Pressure Linked Equations (SIMPLE). Hence, a result comparison of Xenos and Tzirtzilakis’s findings could prove the applicability of the developed source code. If through the implementation of different numerical techniques, similar results could be achieved, then the algorithms would be confirmed to be working properly.

First, a mesh dependency test was performed to ensure the results obtained would have meshed independence. To compute the solutions, the following parameters were used, such that Re = 400, *ρ* = 1050kgm^−3^ and *m* = *μ* = 0.0035kgm^−1^s^−1^. For validation purposes, the system of equations governing fluid flow in Comsol Multiphysics and Matlab software were prescribed with boundary conditions for the stated computational domain following the one implemented in [39]. The flows were governed by similar governing equations as specified in Eq 14, subjected to the following working conditions being imposed along the boundary of the domain:

$$\begin{array}{c} \begin{array}{cc} & \text{Inflow:}\;u\left(x,y\right)=1-{\left(\frac{y}{0.5}\right)}^{2},\;v\left(x,y\right)=0,\;\text{at}\;\left(x=0,-0.5\leq y\leq 0.5\right),\;\\ & \text{Outflow:}\;\left(-pI+\frac{1}{\text{Re}}2\eta \left(\dot{\gamma }\right)D\right)n=t_{h}\;\text{on}\;\Gamma_{h},\\ & \text{Top}\;\text{and}\;\text{bottom}\;\text{wall:}\;u=0,\;\text{on}\;\Gamma_{g}. \end{array} \end{array}$$ (29)

**Fig 2:**
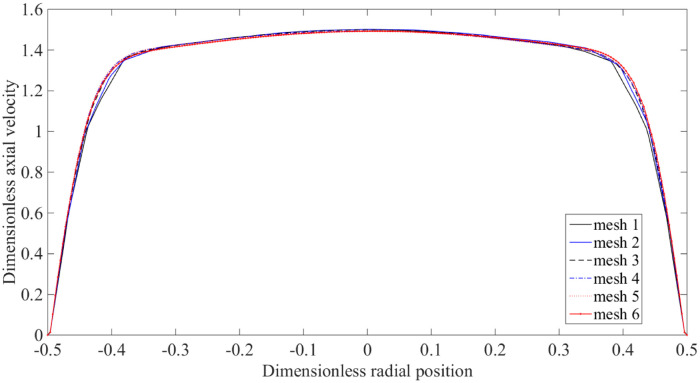
The dimensionless axial velocity, *u* at the centre of the stenosis, *x* = 0 for mesh 1 = 3516, mesh 2 = 7684, mesh 3 = 11780, mesh 4 = 17322, mesh 5 = 23346 and mesh 6 = 25108.

The results generated from several domain elements were then plotted as shown in Fig 2, with each containing 3516, 7684, 11780, 17322, 23346, and 25108 domain elements, respectively. By using several domain elements, the dimensionless axial velocity distributions were evaluated along the centre of the stenosis (*x* = 0). As depicted in Fig 2, the variation between each curve is very small and mesh 4, mesh 5, and mesh 6 are quite similar. This shows that the results are independent of the total number of elements for these three consecutive meshes. Therefore, to gain a sufficient solution for the following problem, the total number of elements needed was 19000-22000, to generate around 10000-12000 nodes containing 29000-33000 total degrees of freedom, resulting in 29000×29000 to 33000×33000 sized global stiffness matrixes and global Jacobian matrixes, respectively. Hence, to provide a good vortex formulation, the computational domain is meshed with 21060 number of triangular elements to approximate a solution for blood flowbehaviour in this section.

By using the selected meshing, the results obtained for maximum velocity with location and pressure drop are displayed in Table 1 for validation purposes. The result obtained from Comsol Multiphysics in Table 1 was solved using 19086 unstructured triangular elements. A comparison of results made with the previous literature and Comsol Multiphysics software showed a very tiny difference between maximum *u*-velocity and location with the one computed in this study. The results were within a satisfactory range, indicating close agreement. Slight differences might have occurred due to differences in mesh sizes, flow unsteadiness, and the use of different methods. On the other hand, the values for pressure drop obtained in this study were found to be in excellent agreement with those reported in [39] as well as the results computed using Comsol Multiphysics software.

**Table 1:** Results for maximum *u*-velocity with its location and pressure drop, ∇*p*.

Results from:	max *u*-velocity with its location	Pressure drop,∇*p*
Present study	1.5993 at (0.286,0)	0.7928
Xenos and Tzirtzilakis [39]	1.5360	0.8
Comsol Multiphysics	1.5785 at (0.2715,-0.0012)	0.7856

**Fig 3:**
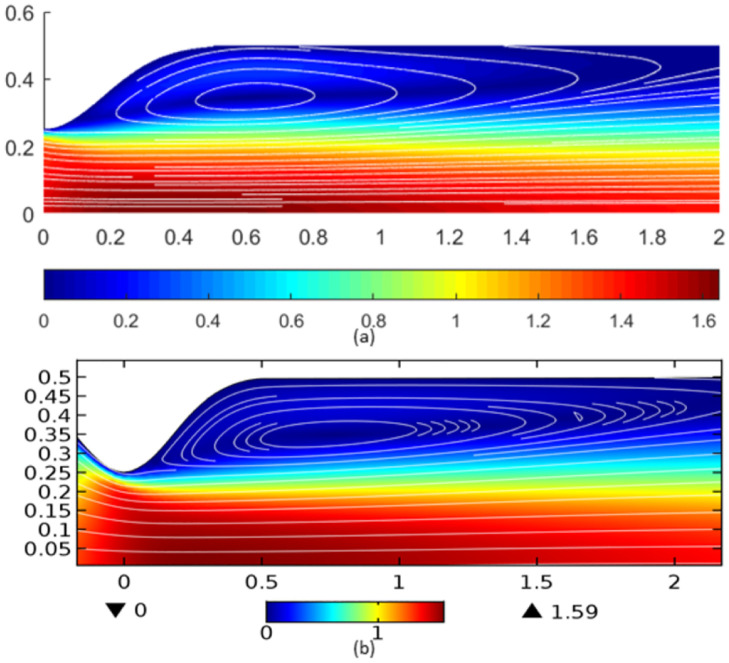
Velocity contour obtained from (a)Present study is comparable with (b)Comsol Multiphysics and [39].

The results for velocity contours and streamline patterns were also demonstrated for validation. As can be seen from Fig 3, the contour for velocity and vortex obtained from this study’s source code were identical to the results of [39] and Comsol Multiphysics.

Overall, from the performed numerical validation, the applicability of the developed algorithms was proven. Thus, it was reliable for solving the problems involved in this particular study, which are further discussed in the next section.

## Results and discussion

This research demonstrates a numerical solution for purely viscous blood flow through a bifurcated artery with overlapping shaped stenosis subjected to an external magnetic field. A thorough quantitative analysis of Galerkin least-squares formulation on the stenosed bifurcated artery flow phenomenon was investigated, where the parameters used were adopted from [8, 37] as:

$$\begin{array}{c} \begin{array}{cc} & a=0.0075\text{m},\;l_{0}=0.015\text{m},\;d=0.005\text{m},\;x_{max}=0.06\text{m},\;x_{1}=0.025\text{m},\\ & \beta =30^{{}^{\circ}},\;q=0.0002\text{m},\;r_{1}=0.51a,\;\bar{h}=2a,\tau_{m}=0.2\text{a}\;\text{and}\;0.4\text{a},\\ & \rho =1050\text{kg}{\text{m}}^{-3},\;\mu =0.0035\text{kg}{\text{m}}^{-1}{\text{s}}^{-1},\;\text{and}\;\sigma =0.8\text{S}{\text{m}}^{-1}. \end{array} \end{array}$$ (30)

**Fig 4:**
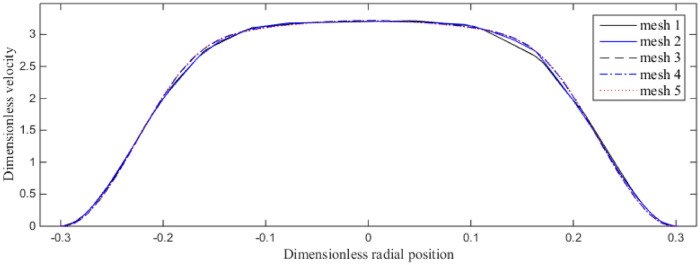
The dimensionless axial velocity, *u* at the second throat of the stenosis, *x* = 1 for mesh 1 = 7787, mesh2 = 10352, mesh 3 = 13998, mesh 4 = 17090 and mesh 5 = 28196.

To confirm that the computed results are independent on the mesh parameters, a mesh independence test was conducted as in the preceding section on the domain of interest, with several mesh numbers as illustrated in Fig 4. The dimensionless axial velocity across the second throat of stenosis (*x* = 1) was computed using a different domain element numbers, which were 7787, 10352, 13998, 17090, and 28196 as visualised in the present figure. A mesh independence test was conducted to determine the most efficient meshing for computing whole solutions. The results in Fig 4 were meshed using unstructured linear triangular elements. Furthermore, numerical integrals were computed using six quadrature points with a convergence tolerance of *τ* = 0.0001. The results published for this test were computed for a Newtonian flow (*n* = 1) of blood through a stenosed bifurcated artery with a stenosis height of *τ*_*m*_ = 0.4*a* using Reynolds number Re = 300.

**Fig 5:**
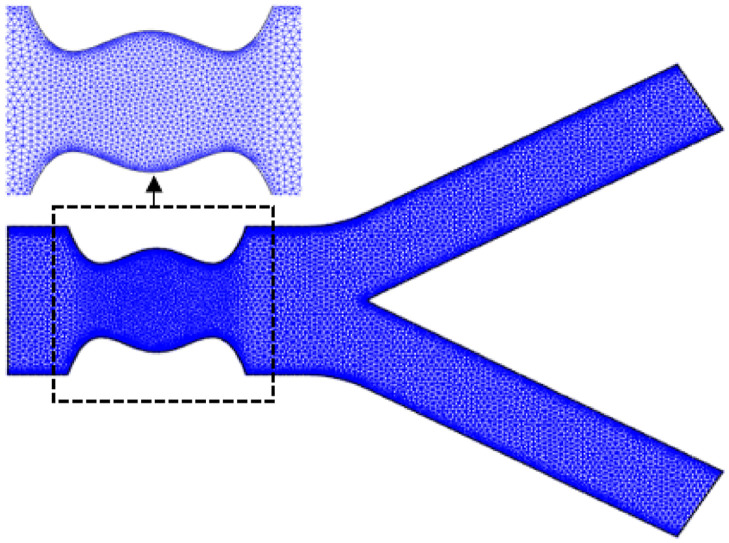
The meshing selected containing 12735 triangular elements for *τ*_*m*_ = 0.4*a*.

As can be seen from Fig 4, the velocity profiles plotted for mesh 2, mesh 3, mesh 4, and mesh 5 are similar and overlap with each other. Therefore, it can be concluded that these four consecutive meshes are independent of the total number of elements. Since the velocity profile plotted using mesh 1 slightly deviates from the other velocity profiles. Hence, to get a satisfactory solution, the total number of elements needed to compute the solution for this type of computational domain is between 11000-13000 with 6000-8000 nodes. This will generate a degree of freedom of around 19000-23000 and a global Jacobian and stiffness matrix sized 19000×19000 to 23000×23000. Using these aforementioned characteristics, Fig 5 illustrates that the selected meshing for the stenosed bifurcated channel contained 12735 unstructured triangular elements constructed from 6880 nodes with a maximum allowed element size of 0.03. It is worth noting that the global element matrix and the global tangent matrix generated using the selected mesh have a dimension of 12735×12735 with a 12735 global degree of freedom. The meshing for the computational domain was refined along the stenotic region, since those sites experienced a significant change in blood flow characteristics. In this section, the effects of a transverse magnetic field on the haemodynamic changes of blood flow involving flow velocity, pressure, wall shear stress, and streamline pattern were thoroughly examined. Shear-thickening and shear-thinning effects were demonstrated by representing those streaming fluids according to the generalised power law index of *n* = 1.2 and *n* = 0.639, respectively. At *n* = 1, the blood rheology was treated as a Newtonian fluid with a constant fluid viscosity, *μ* throughout the vessel.

**Fig 6:**
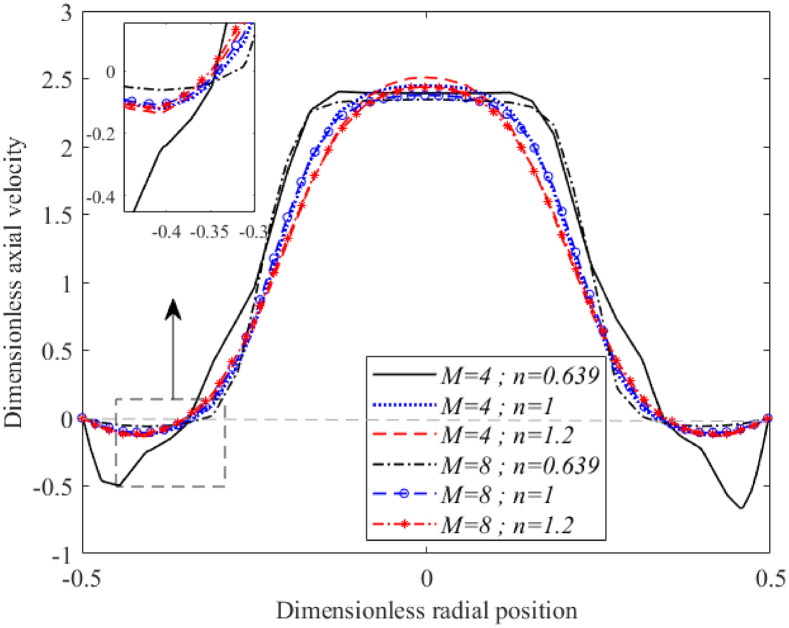
Variation of axial velocity at *x* = 1.5 with different in Hartmann number and fluid characterisation (Re = 300 and *τ*_*m*_ = 0.4*a*).

As shown in Fig 6, the effects of different Hartmann numbers on shear-thinning, Newtonian, and shear-thickening fluids axial velocities at the post-stenotic region (*x* = 1.5) with 40% severity are depicted for Re = 300. The axial velocity profiles for shear-thinning, Newtonian, and shear-thickening fluids exhibited the highest peak velocity in the lowest magnetic field intensity where *M* was considered to be 4. As the MHD effect with higher intensities of *M* = 8 were applied to fluid flow, a small drop in the dimensionless axial velocities at the centre of an artery was observed in all fluids. In contrast, with increases in magnetic field intensity, velocity near the arterial wall increased, preventing the formation of flow reversal for all fluids. The shape of the profile attained by the shear-thickening fluid was more parabolic and more restricted towards the negative flow than its shear-thinning and Newtonian fluid counterparts. The shape of the profile attained by the Newtonian flow (*n* = 1) serves as a transition from the flat shape obtained by the shear-thinning fluid (*n* = 0.639) to a more parabolic shape shown by the shear-thickening fluid (*n* = 1.2).

**Fig 7:**
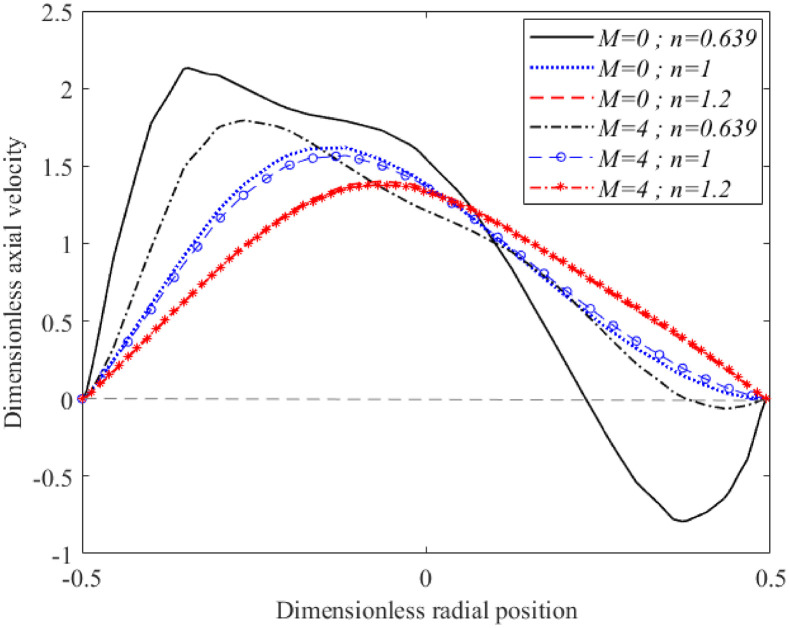
Variation of axial velocity at *x* = 3.0118 with different in Hartmann number and fluid characterisation (Re = 300 and *τ*_*m*_ = 0.4*a*).

The effects of the Hartmann number on the flow velocity at the daughter branch was studied using different fluid characterisations as illustrated in Fig 7. The fluids were set to flow through a bifurcated artery that possessed overlapping stenosis with 40% occlusion located at the parent’s arterial lumen. There was no flow reversal spotted over this region for the Newtonian and shear-thickening fluids in the presence and absence of a magnetic field. The shear-thinning fluid on the other hand formed a negative flow region in the vicinity of the outer arterial wall that was considerably reduced as magnetic field intensity increased from *M* = 0 to *M* = 4. All of the curves skewed to the right after passing through a negative flow area that formed in the post-stenotic region of the outer arterial wall. With increased magnetic field intensity, the flow at the daughter branch decelerated appreciably. Misra and Shit [10] found that arteries with smaller diameters were slightly affected by magnetic field intensity. Fig 7 shows that the amount of decrement in the region that did not possess any arterial lumen constriction was larger than the one observed in Fig 6, which consisted of stenosis with a 40% occluded region. Hence, this finding is in agreement with results reported in [10]. Besides that, from the comparison of velocity plotted in Figs 6 and 7, velocity at the parent artery is higher than the fluid’s velocity at the daughter branch. Similar findings were reported in [43]. The presence of arterial constriction at the parent artery significantly hindered the flow of blood irrespective of fluid nature not only along the stenotic region but also downstream of it as shown in the current figure where the magnitude of the flow’s velocity dropped considerably in comparison to Fig 6.

**Fig 8:**
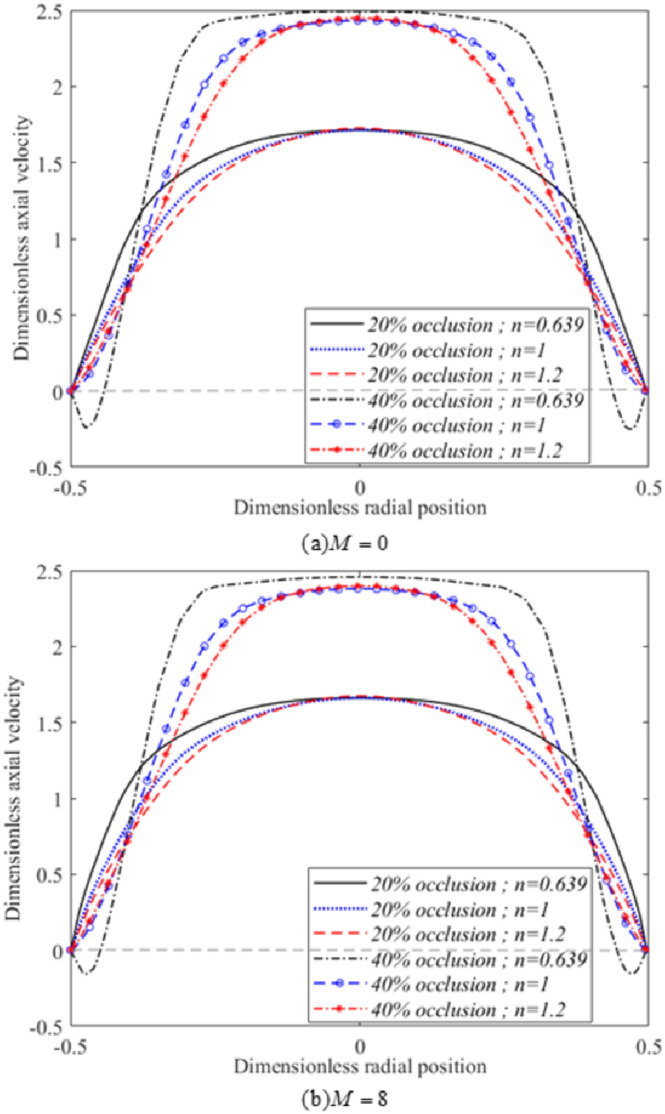
Variation of axial velocity with different stage of stenosis severity and Hartmann number at *x* = 1 (Re = 300) for: (a)*M* = 0 and (b)*M* = 8.

The relation between the severity of stenosis and magnetic field intensity for different fluids was later clarified by the curves representing the axial velocity profiles across the second throat of stenosis shown in Fig 8a and 8b. As can be seen, increases in stenosis size considerably enhanced flow velocities at the throat of the stenosis, irrespective of fluid characterisation. Flow reversal was found to develop for shear-thinning fluids with 40% occlusion at *M* = 0 and 8. As magnetic field intensity was raised to *M* = 8, flow reversal decreased. The increase in magnetic field intensity did not significantly change the maximum values for the axial velocity profiles at the centre of this region. Somehow, a significant relationship between magnetic field intensity and axial velocity for the three fluid types was observed at the vicinity of the arterial wall. The application of magnetic fields managed to extensively overcome the formation of flow reversal in the vicinity of the arterial wall. The fluid flow at the centre of the vessel has slightly reduced for the three fluids as magnetic field intensity increased.

**Fig 9:**
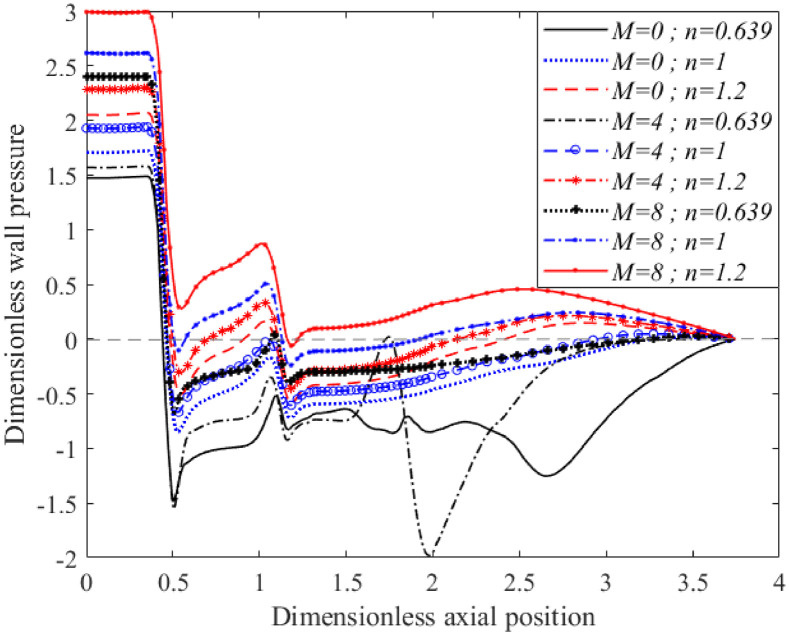
Wall pressure distribution along the outer arterial wall for different in Hartmann number and fluid characterisation (Re = 300 and *τ*_*m*_ = 0.4*a*).

The study of pressure loss is crucial since it can be an indication of adequate blood supply to the organs. Hence, as shown in Fig 9, wall pressure along the outer wall for the non-Newtonian model for the shear-thickening, Newtonian, and shear-thinning blood flows through a bifurcated artery with 40% occlusion were plotted according to different Hartmann numbers, *M* = 0, 4, and 8. All the curves showed a similar pattern from the arterial’s inlet until the offset of the stenosis, in with the shear-thickening fluid showed higher pressure values than its counterparts. The wall pressure in Fig 9 also showed a rapid drop at the first throat of overlapping stenosis, resulting from a sudden change in arterial radii that had accelerated the flow through an arterial constriction. The shear-thinning fluid exhibited a significant fluctuation of blood pressure along the outer arterial wall after passing through the stenotic region, which was predominantly observed for magnetic field intensities of *M* = 0 and 4. As a magnetic intensity slightly higher than *M* = 8 was applied, the fluctuated blood pressure of the shear-thinning fluid was overcome, in that it retained the pressure profile of its counterparts. The lowest wall pressure values for shear-thickening and Newtonian fluid were found at the offset of the overlapping stenosis, indicated by negative values for *M* = 0, 4, and 8. The shear-thinning fluid exhibited negative values at its lowest peak, which formed at the curvature junction before entering the daughter branch wall for *M* = 0 and 4. The negative pressure values exerted on the wall, especially the one formed at the stenotic region, might have caused the artery to collapse due to insufficient pressure to support the opening of the arterial lumen.

**Fig 10:**
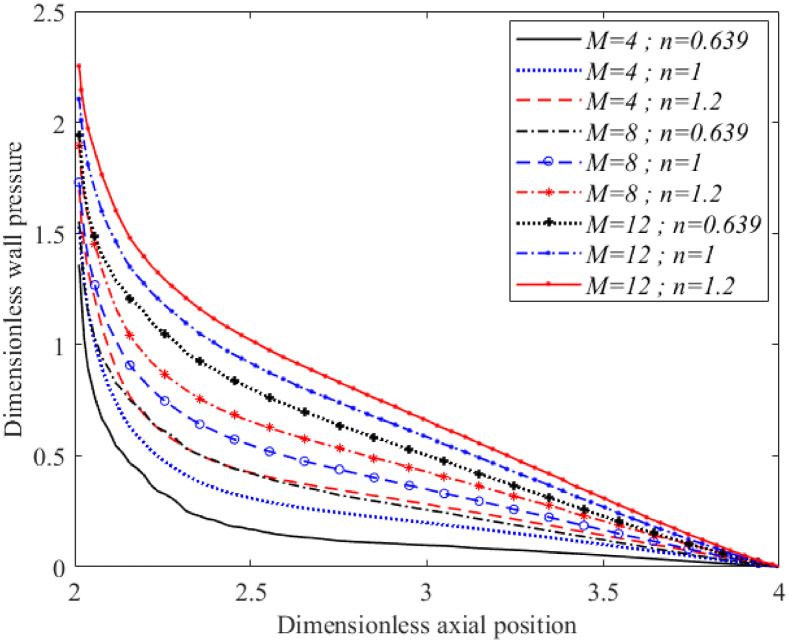
Wall pressure distribution along the inner arterial wall for different in Hartmann number and fluid characterisation (Re = 300 and *τ*_*m*_ = 0.2*a*).

Similar to Figs 9 and 10 shows that pressure distribution along the inner wall of the bifurcated artery with 20% occlusion area on the stenotic region also increased with increased Hartmann numbers, regardless of fluids characterisation. All the curves gradually decreased from their respective maximum value at the apex to a zero pressure at the outlet. There was no negative value along the inner wall of the branch artery in the presence of a magnetic field. The pressure values along the inner arterial wall for the shear-thickening fluid were observed to be greater than the Newtonian and shear-thinning fluid. The shear-thickening fluid possessed a more viscous flow, which exerted greater friction between the fluid layers and the arterial wall. The pressure exerted on the wall resulted from normal stress that was perpendicular to the vessel wall.

**Fig 11:**
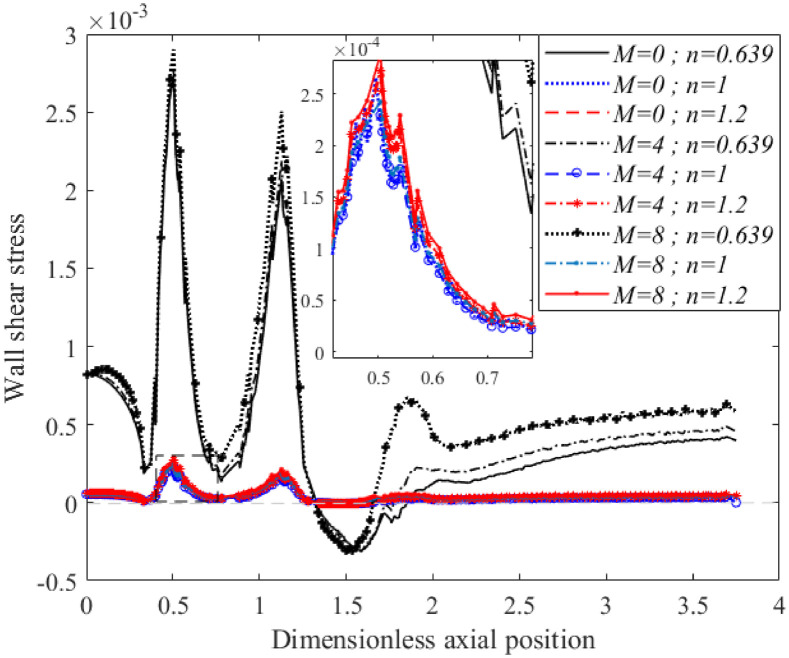
Wall shear stress along the outer arterial wall for different in Hartmann number and fluid characterisation (Re = 300 and *τ*_*m*_ = 0.2*a*).

Shear stress was the second force component exerted on the wall, which was exhibited by a tangential stress from blood viscosity. The friction between the blood and the vessel wall created a velocity gradient (shear rate) that varied along the arterial wall. The variations in strain rate times the molecular fluid viscidity as calculated in Eq 28 were used to determine variations in shear stress [43]. From the computed values, the progress of arterial diseases can be predicted. Due to its significance to blood flow studies, as shown in Fig 11, the distribution of shear stress along the outer arterial wall was demonstrated for pseudoplastic, Newtonian and dilatant fluids that passed through a bifurcated artery with 20% occlusion at the stenotic region. The shear-thinning, Newtonian, and shear-thickening fluids showed similar distribution of wall shear stress, with peaks developed in the extremities of the first and second throat of stenosis which agreed qualitatively with stenotic blood flow studies by [8]. The steep enhancements that occurred near the throats of the overlapping stenosis might have promoted platelet activation, which could have induced thrombosis [8]. The magnitude of wall shear stress for the shear-thinning fluid were greater than the shear-thickening and Newtonian fluids at most spatial locations, with a major difference occurring in the stenotic region. This happened due to the sharper velocity gradients (shear rate) and accelerated flow characterised by the shear-thinning fluid at the constricted area. However, the influence of MHD appeared to have a minor effect on the site where stenosis was located. This was due to a smaller arterial diameter along the stenotic region, which reduced the efficiency of the magnetic field [10].

Accurate estimations of wall shear stress distribution are significant for atherosclerosis risk predictions. From the estimated values for this haemodynamic factor, the influence of blood flow through a diseased vessel on endothelial cells can be understood. For instance, low wall shear stress values are the favoured site of atherosclerotic plaque development, while the high shear stress regions that developed in the extremities of the stenosis throat may contribute to platelet activation, which may destroy the vessel wall and contribute to the formation of intimal thickening [44]. High shear values may also induce thrombosis and block the flow of blood [44]. Based on that clinical evidence, the flow of blood corresponding to shear-thinning fluids shows a higher risk of platelet activation, which may contribute to thrombosis at the constricted region in comparison to the Newtonian and shear-thickening fluids. Also, the shear-thinning fluid showed a large low wall shear stress region downstream of the constriction, which could degrade the arterial wall and lead to worse atherosclerosis conditions in comparison to its counterparts. The distributions of wall shear stress at the branch artery presented here agreed qualitatively with the one published in [37, 45].

**Fig 12:**
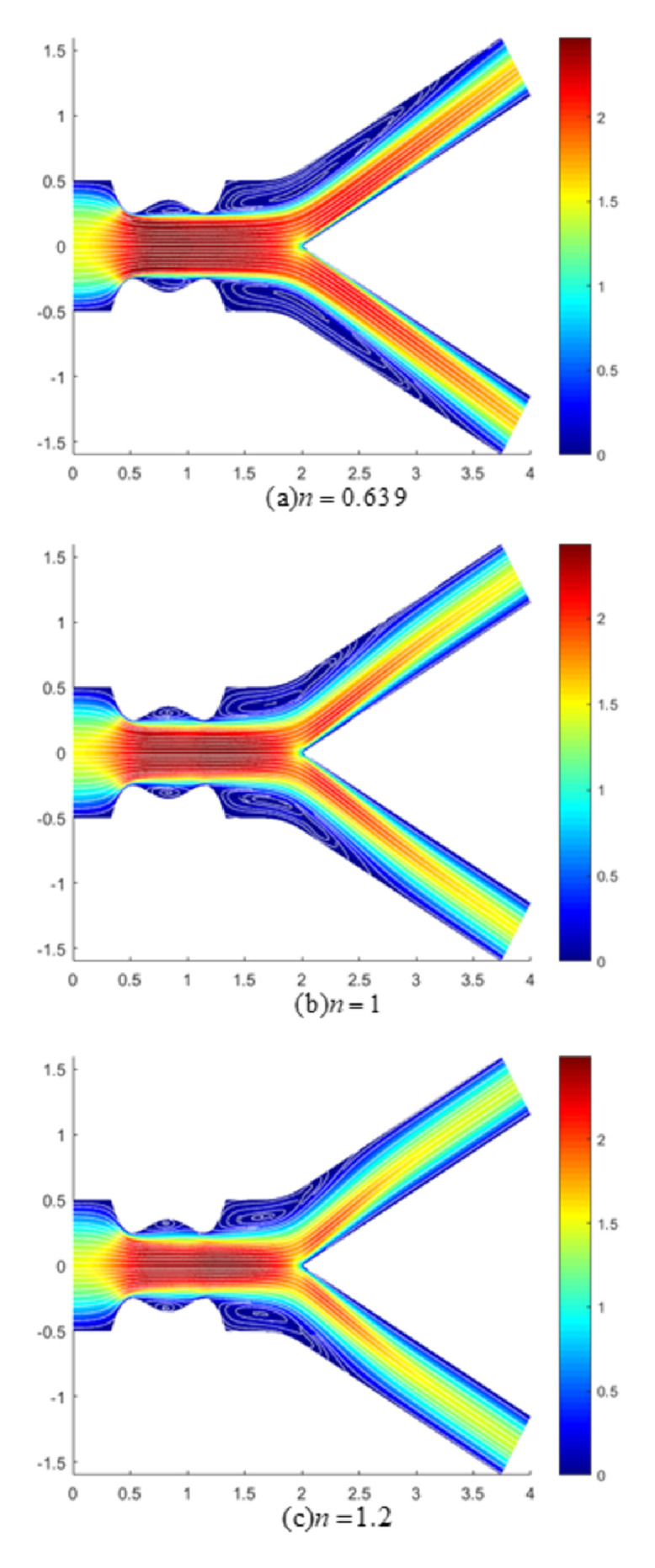
Velocity contour with streamlines pattern for (a)*n* = 0.639 (shear-thinning), (b)*n* = 1 (Newtonian) and (c)*n* = 1.2 (shear-thickening) fluids (*M* = 8, Re = 300 and *τ*_*m*_ = 0.4*a*).

**Fig 13:**
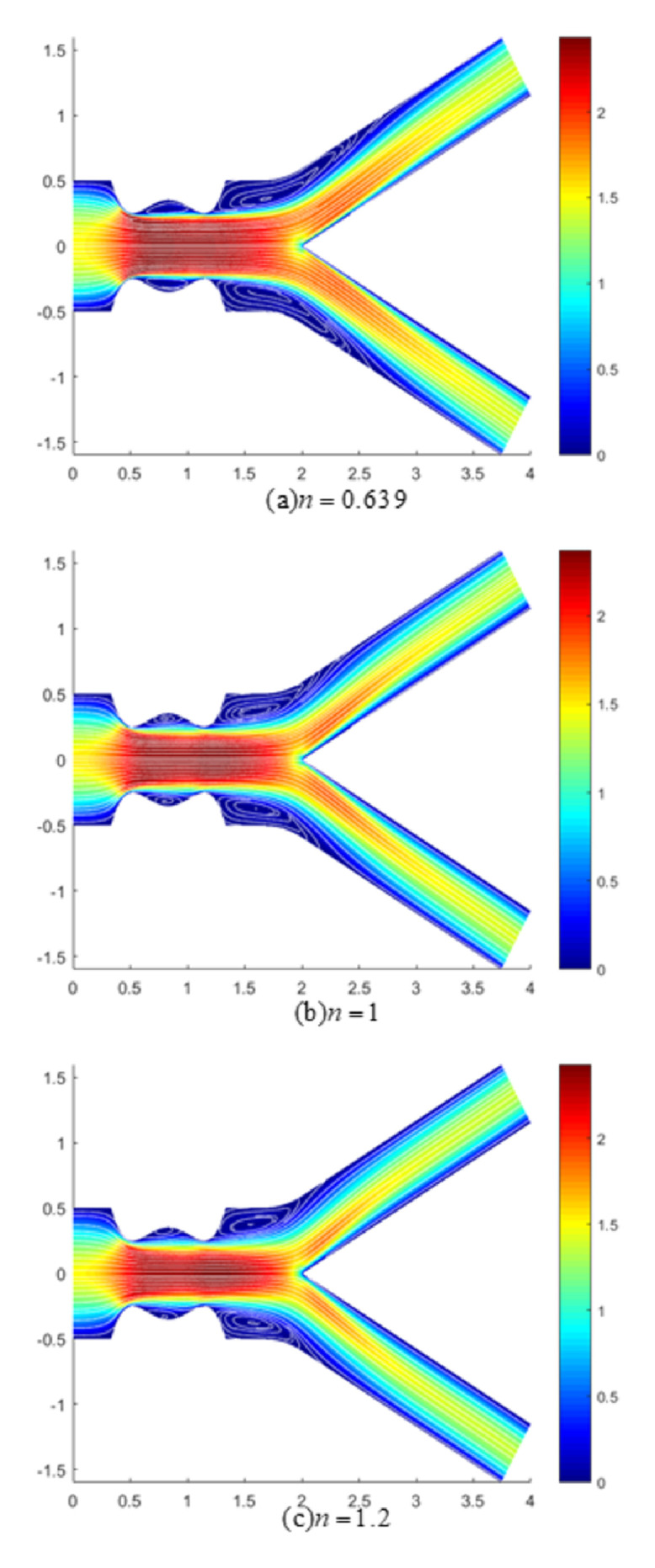
Velocity contour with streamlines pattern for (a)*n* = 0.639 (shear-thinning), (b)*n* = 1 (Newtonian) and (c)*n* = 1.2 (shear-thickening) fluids (*M* = 12, Re = 300 and *τ*_*m*_ = 0.4*a*).

The accumulation of plaque reduced the arterial passage area, complicating the transport of blood across this area through the formation of flow reversal and recirculation zones downstream of the stenosis. In view of this, the nature of streamlines in the presence of a magnetic field at Re = 300 was visualized with velocity contours in Figs 12 and 13 for an arterial bifurcation with 40% stenosis for shear-thinning, Newtonian and shear-thickening fluids at *M* = 8 and 12, respectively. With increased magnetic field strengths, a substantial reduction of recirculation zones was noticed, with the largest vortexes being formed at low magnetic field intensities (*M* = 8). Also, from the comparison between the recirculation zones exhibited by shear-thinning, Newtonian, and shear-thickening fluids at constant magnetic field intensity, it can be seen that the shear-thinning fluid formed longer eddies than its counterparts. The shortest eddies were developed by the shear-thickening fluid with the Newtonian fluid developing eddies with intermediate lengths between the shear-thinning and shear-thickening fluids. This is because the shear-thinning fluid was less viscous at the low shear rate region, thus having a greater velocity, causing the fluid layers to be less attached to the arterial walls. At much higher magnetic intensities (*M* = 12), it was noticed that the vortex emerging downstream of the stenotic region was significantly reduced in size for the three fluids, producing a uniform calm flow that reduced the risk of thrombosis along the stenotic region.

Based on the velocity contour, an accelerated flow of blood was developed along the occluded region. It can be clearly observed from the contour colours that the shear-thinning fluid exhibited a larger flow with lower velocity downstream of the stenotic region in the vicinity of the arterial wall compared to the Newtonian and shear-thickening fluids, indicating the occurrence of flow stagnation in that region. Besides, with increased magnetic intensities, each fluid flow at the daughter branches decreased. The drop in blood flow to the daughter branches can lead to an insufficient supply of blood to organs and tissues, which may contribute to worsening individual health. The flow disturbance that predominantly developed at the downstream region of the stenosis on the other hand activated the coagulation system, which resulted in the production of a fibrin-rich tail of thrombus [46].

## Conclusion

In this study, a numerical simulation of magnetohydrodynamic blood flow through a stenosed bifurcated artery was successfully conducted using the Galerkin least-squares method. By representing the generalised power law index, *n* according to various blood flow natures of *n* = 0.639 for shear-thinning fluid, *n* = 1 for Newtonian fluid, and *n* = 1.2 for shear-thickening fluid, the effects of different fluids characterisations through a bifurcated artery on blood behaviour was thoroughly examined. This study is particularly applicable to a situation where the arterial lumen of the blood vessel is deposited with plaque, which in this study was modelled as overlapping stenosis. The nonlinear governing equations were solved numerically with the help of the Newton-Raphson method. The results presented from this work show important information regarding the complex behaviour of blood in situations, where the physical parameters *M*, *n*, *Re* and *τ*_*m*_ play a prominent role in haemodynamic flow subjected to the application of a uniform magnetic field in the transverse direction. The findings from this study on non-Newtonian fluid theory will help in understanding the haemodynamic factors that influence the rheological behaviour of blood flows. The computed results may also provide information on flow disturbances that might develop due to stenosis. This investigation is beneficial for medical practitioners in analysing the impact of magnetic fields on patients during magnetic therapy. This study is also beneficial for the design of future medical devices that utilise magnetic fields. The key findings of the study are based on numerical results reported in the preceding sections:

1. The rise in Hartmann number will cause the velocity of fluids to decelerate, irrespective of different fluid natures. The axial velocity, which decreases at the centre and increases near the vessel wall will restrict the occurrence of flow reversal. MHD effects are proven to significantly affect the flow velocity at regions with no constriction, rather than regions that possesses a constriction.
2. The rise in Hartmann number and generalised power law index leads to significant drops in pressure. Also, increasing magnetic intensity will improve the negative pressures exerted on walls downstream of the stenotic region, as well as at the constricted region itself for the three fluids.
3. Recirculation areas were reduced with increases in the Hartmann number, indicated by the percentage reduction in recirculation regions of about 39% for a shear-thinning fluid, 26% for a Newtonian fluid, and 27% for a shear-thickening fluid as magnetic intensity was enhanced from *M* = 8 to *M* = 12. The shear-thinning fluid exhibited a larger recirculation area in comparison to the Newtonian and shear-thickening fluids.
4. The flow disturbances that occurred mostly along the downstream region from the stenosis did have a significant impact on the transport of blood into the daughter branches since fluid velocity dropped appreciably at the daughter branch in comparison to the mother artery. It is worth noting that the enhancement of magnetic intensity from *M* = 0 to *M* = 4 contributed to a drop in average flows velocity at the daughter artery by 0.0449, 0.0081 and 0.0011 for the shear-thinning, Newtonian, and shear-thickening fluids, respectively. Whereas, the average velocity for the shear-thinning, Newtonian, and shear-thickening fluids at the second throat of overlapping stenosis located at the parent artery were decreased by 0.0025, 0.0017 and 0.0012, respectively, as *M* was increased from 0 to 4.
5. The great wall shear stress in the extremities of an overlapping stenosis throat were reduced with increased magnetic field intensities. The wall shear stress recorded for the shear-thinning fluid was greater than the shear-thickening and Newtonian fluids at all spatial locations except for the downstream region of stenosis prior to the daughter branch. Due to this, this shear-thinning fluid possesses a higher risk of platelets activation, which may induce thrombosis and lead to the worsened or total blockage of blood flow.

This matter highlights the importance of using the power law index in blood flow studies to describe various non-Newtonian natures corresponding to the rheological behaviour of blood under diseased conditions. In addition, the consideration of arterial bifurcation as the computational domain in this study was essential since this site is prone to atherosclerosis. The results in this study from the use of the GLS method offers an alternative to the usual FEM in solving shear flow through a complex domain, which have failed to be solved using the standard Galerkin FEM. This method enhances the convergence of solutions without affecting their consistency by introducing a stabilization parameter that is mesh-dependent.

## Abbreviations

*B*_*o*_: Magnetic flux intensity (T)
**D**: Strain rate tensor
**f**: Body force vector
FEM: Finite Element Method
GLS: Galerkin Least-Squares
GNL: Generalized Newtonian Liquid
$\bar{h}$: Length of the inlet (m)
**I**: Unit tensor
*I*_2_: Second Invariant of strain rate tensor
*m*: Fluid consistency (kgm^−1^s^−1^)
M: Hartmann number (Dimensionless)
*n*: Power law index
**n**: Unit outward normal vector
$\bar{p}$: Pressure (kgm^−1^s^−2^)
Re: Reynolds number (Dimensionless)
**t**_*h*_: Boundary tractions vector
$\bar{u}$: Velocity vector (ms^−1^)
*u*: *x*-velocity (Dimensionless)
${\bar{u}}_{r}$: Average mean inflow velocity (ms^−1^)
*v*: *y*-velocity (Dimensionless)
*x*: *x*-coordinates (Dimensionless)
*y*: *y*-coordinates (Dimensionless)
$\dot{\gamma }$: Magnitude of strain rate tensor
Γ_*g*_: Boundary of Dirichlet conditions
Γ_*h*_: Boundary of Neumann conditions
$\eta (\dot{\gamma })$: Effective viscosity function (Dimensionless)
*μ*: Viscosity of Newtonian model (kgm^−1^s^−1^)
*ρ*: Density of blood (kgm^−3^)
*σ*: Electrical conductivity of fluid (Sm^−1^)
Ω: Domain
